# Chromosomal abnormalities detected by chromosomal microarray analysis and pregnancy outcomes of 4211 fetuses with high-risk prenatal indications

**DOI:** 10.1038/s41598-024-67123-5

**Published:** 2024-07-10

**Authors:** Huafeng Li, Juan Hu, Qingyu Wu, Jigang Qiu, Li Zhang, Jinping Zhu

**Affiliations:** Genetic Medical Center, Women and Children’s Health Care Hospital of Linyi, Liyin, 276014 China

**Keywords:** Prenatal diagnosis, Chromosomal microarray analysis, Copy number variations, Pregnancy outcomes, Genetics, Chromosomes

## Abstract

With the gradual liberalization of the three-child policy and the development of assisted reproductive technology in China, the number of women with high-risk pregnancies is gradually increasing. In this study, 4211 fetuses who underwent chromosomal microarray analysis (CMA) with high-risk prenatal indications were analysed. The results showed that the overall prenatal detection rate of CMA was 11.4% (480/4211), with detection rates of 5.82% (245/4211) for abnormal chromosome numbers and 5.58% (235/4211) for copy number variants. Additionally, the detection rates of clinically significant copy number variants were 3.78% (159/4211) and 1.8% (76/4211) for variants of uncertain significance. The detection rates of fetal chromosomal abnormalities were 6.42% (30/467) for pregnant women with advanced maternal age (AMA), 6.01% (50/832) for high-risk maternal serum screening (MSS) results, 39.09% (224/573) with abnormal non-invasive prenatal testing (NIPT) results, 9.21% (127/1379) with abnormal ultrasound results, and 5.1% (49/960) for other indications. Follow-up results were available for 4211 patients, including 3677 (3677/4211, 87.32%) whose infants were normal after birth, 462 (462/4211, 10.97%) who terminated their pregnancy, 51 (51/4211, 1.21%) whose infants were abnormal after birth, and 21 (21/4211, 0.50%) who refused follow-up. The results of this study demonstrate significant variation in the diagnostic rate of chromosomal microarray analysis across different indications, providing valuable guidance for clinicians to assess the applicability of CMA technology in prenatal diagnosis.

## Introduction

China has a large population and ranks among the countries with a high prevalence of birth defects. In China, there are significant disparities among different geographical regions, with the prevalence of birth defects ranging from 0.715 to 19.184%^1–3^. In addition to traditional chromosomal aneuploidy abnormalities, chromosome microdeletion or microduplication syndromes have emerged as a significant category of birth defects. The diagnostic rate of chromosomal microarray analysis (CMA) for chromosomal abnormalities is higher than that of traditional G-banding karyotype analysis. The prenatal diagnosis of microdeletion and microduplication syndrome through CMA has significant implications for the prevention of birth defects.

The indications for invasive prenatal diagnosis mainly include abnormal ultrasound findings, high-risk maternal serum screening (MSS), abnormal non-invasive prenatal testing (NIPT) results, advanced maternal age (AMA), intrauterine growth restriction, history of adverse pregnancy outcomes, maternal request, in vitro fertilization, drug use or exposure to toxic substances during pregnancy, consanguineous marriage, and parental anxiety. Since 2009, the United States, Europe, Canada, and other developed countries have recommended CMA as the first-tier diagnostic test for fetuses, both for patients with fetal ultrasound anomalies and for patients who choose to undergo prenatal testing despite normal ultrasound of the fetus^4–6^. To date, many studies evaluating the performance of CMA in prenatal diagnosis have been published^7–9^. The incidence of pathogenic CNVs in fetuses with abnormal ultrasound results can be further classified based on the specific organ system involved and the number of observed abnormalities. The most frequently affected organ systems related to abnormal CMA results are the cardiovascular system, skeletal system, genitourinary system and central nervous system^10–13^. However, limited knowledge exists regarding the incidence of clinically significant CNVs in fetuses with structural abnormalities of the anatomical system or ultrasound soft marker abnormalities. Moreover, limited information is available regarding the diagnostic advantages of CMA for pregnant women opting for invasive prenatal testing due to other indications such as AMA, abnormal MSS results and abnormal NIPT results. Given China's large population, vast territory, and uneven economic development level, a comprehensive understanding of the application of CMA technology among high-risk groups in specific regions would contribute to strengthening the popularization of this technology and government support policies. Therefore, we conducted a comprehensive and systematic analysis of CMA test results from 4211 high-risk pregnant patients to determine the incidence of CMA abnormalities detected across different indications. This study aimed to provide guidance for clinicians in evaluating the applicability of CMA technology for prenatal diagnosis and the prevention of birth defects.

## Results

### Demographic data

From April 2016 to December 2022, 4211 women underwent invasive prenatal genetic testing at our center. Among them, 169 patients (169/4211, 4%) underwent chorionic villus biopsy, while 4042 patients (4042/4211, 96%) underwent amniocentesis. Clinical indications included only advanced maternal age (467/4211, 11.09%), high-risk maternal serum screening results (832/4211, 19.76%), abnormal NIPT results (573/4211, 13.6%), abnormal ultrasound results (1379/4211, 32.75%) and other indications (960/4211, 22.80%), including intrauterine growth restriction, history of adverse pregnancy outcomes, maternal request, in vitro fertilization, drug use or exposure to toxic substances during pregnancy, consanguineous marriage, and parental anxiety. The mean gestational age was 12 weeks (11–13) for patients who underwent chorionic villus biopsy and 20 weeks (16–30) for patients who underwent amniocentesis.

From April 2016 to December 2022, a total of 4211 samples were analysed by CMA. Among these, 480 samples (480/4211, 11.4%) were found to be abnormal, including 245 (245/4211, 5.82%) with chromosomal numerical abnormalities and 235 (235/4211, 5.58%) with CNVs (see Table 1). Among the 245 patients with chromosomal numerical abnormalities, 65 (65/4211, 1.54%) had trisomy 21, 29 (29/4211, 0.69%) had trisomy 18, 3 (3/4211, 0.07%) had trisomy 13 (3/4211, 0.07%), 137 (137/4211, 3.26%) had sex chromosome aneuploidy and 11 (11/4211, 0.26%) had rare autosomal abnormalities. Among the 11 patients with rare autosomal abnormalities, 3 had trisomy 2 mosaicism, 2 had trisomy 9 mosaicism, 2 had trisomy 16 mosaicism, 1 had trisomy 22 mosaicism, 1 had trisomy 7 mosaicism, 1 had trisomy 4 mosaicism, and 1 case had monosomy 13 mosaicism. Among the 235 fetuses with CNVs (P/LP/VUS), CMA analysis was performed on 64 parents (64/235, 27.2%). Of these, 47 patients (47/235, 20%) had parental inheritance, while 17 patients (17/235, 7.2%) had de novo mutations. A total of 235 CNVs were identified, with 159 cases (159/4211, 3.78%) classified as pathogenic/likely pathogenic (P/LP) (see Schedule 1), and 76 cases classified as variants of uncertain significance (VUS) (76/4211, 1.80%) (see Schedule 2). Among the different clinical diagnosis groups, the detection rate of chromosomal abnormalities in the group with abnormal NIPT results was significantly higher than that in the other four clinical indication groups (p < 0.001). The detection rate of chromosomal abnormalities in patients with abnormal ultrasound findings is significantly higher than that in patients with other indications (9.21% vs 5.10%, p < 0.001). The differences observed between the other two groups were not statistically significant (p > 0.05).

**Table 1:** CMA results of 4211 samples with different indications for prenatal diagnosis.

Subgroups	Total	Normal	Abnormal	Common autosomal aneuploidies			Rare autosomal aneuploidy	Sex chromosome aneuploidy	CNVs (P/LP)	CNVs (VUS)	
				T21	T18	T13					
AMA- only	467 (11.09%)	437 (93.58%)	30 (6.42%)	6 (1.28%)	2 (0.43%)			2 (0.43%)	11 (2.35%)		9 (1.93%)
Abnormal MSS	832 (19.76%)	782 (94.00%)	50 (6.00%)	6 (0.72%)	5 (0.6%)		2 (0.24%)	3 (0.36%)	21 (2.52%)		13 (1.56%)
Abnormal NIPT	573 (13.60%)	349 (60.91%)	224 (39.09%)***	16 (2.79%)	3 (0.53%)	2 (0.35%)	7 (1.22%)	122 (21.29%)	49 (8.55%)		25 (4.36%)
Abnormal ultrasound	1379 (32.75%)	1252 (90.79%)	127 (9.21%)	34 (2.47%)	17 (1.23%)	1 (0.07%)	1 (0.07%)	8 (0.58%)	49 (3.55%)		17 (1.24%)
Others	960 (22.80%)	911 (94.90%)	49 (5.10%)	3 (0.31%)	2 (0.21%)		1 (0.1%)	2 (0.21%)	29 (3.02%)		12 (1.25%)
Total	4211	3731 (88.6%)	480 (11.4%)	65 (1.54%)	29 (0.69%)	3 (0.07%)	11 (0.26%)	137 (3.26%)	159 (3.78%)		76 (1.8%)

***The significance is at the p < 0.001 level.

### AMA only

The pregnant women in this cohort were aged over 35 years and exhibited no other indications, with an average age of 39.3 years (35–53 years old). There were 30 (30/467, 6.42%) patients whose fetuses had chromosomal abnormalities detected by CMA, including 10 with aneuploidy (10/467, 2.14%) and 20 (20/467, 4.28%) with CNVs. A total of 467 patients of AMA were categorized into three subgroups according to maternal age (see Table 2), and the relationship between maternal age and the chromosomal abnormality rate was analysed. The aneuploidy rate was positively correlated with advancing age, while CNVs did not demonstrate such an increasing trend.

**Table 2:** CMA results of 467 samples with only AMA.

Subgroups	35–39	40–44	≥ 45	Gamma value	p
Normal (437)	229 (93.85%)	182 (93.33%)	26 (92.86%)	–	–
Abnormal (30)	15 (6.15%)	13 (6.67%)	2 (7.14%)	0.047	0.866
Aneuploidy (10)	3 (1.23%)	6 (3.08%)	1 (3.57%)	0.392	0.289
CNVs (P/LP) (11)	7 (2.87%)	4 (2.05%)	0 (0%)	− 0.26	0.878
CNVs (VUS) (9)	5 (2.05%)	3 (1.53%)	1 (3.57%)	− 0.009	0.608

### Abnormal MSS results

The center screened the serum of pregnant women in the second trimester by detecting the levels of AFP and free β-HCG. CMA analysis revealed that 50 of the 832 patients had abnormal MSS results (50/832, 6.00%), including 16 with aneuploidy (16/832, 1.92%) and 34 with CNVs (34/832, 4.08%). The 832 patients with abnormal MSS results were categorized into three subgroups according to risk type: 721 patients with a high risk of trisomy 21 syndrome (721/832, 86.66%), 28 patients with a high risk of trisomy 18 syndrome (28/832, 3.36%) and 83 patients with a moderate risk of trisomy 21 syndrome (83/832, 9.98%). Among them, 6 patients were diagnosed with trisomy 21 syndrome and included in the high-risk group for trisomy 21. There were 5 patients with trisomy 18 syndrome, 4 of whom had a high risk of trisomy 21 syndrome and 1 who had a high risk of trisomy 18 syndrome (see Table 3).

**Table 3:** CMA results of 832 samples with abnormal MSS.

Subgroups	Total	Normal	Abnormal	T21	T18	Sex chromosome aneuploidy	Rare autosomal aneuploidy	CNVs (P/LP)	CNVs (VUS)
High risk of trisomy 21 syndrome (≥ 1/270)	721 (86.66%)	678 (94.04%)	43 (5.96%)	6(0.83%)	4 (0.55%)	2 (0.28%)	2 (0.28%)	19 (2.63%)	10 (1.39%)
High risk of trisomy 18 syndrome (≥ 1/350)	28 (3.36%)	25(89.29%)	3 (10.71%)		1 (3.57%)	1 (3.57%)			1 (3.57%)
Intermediate risk of trisomy 21 syndrome (1/270–1/1000)	83 (9.98%)	79 (95.18%)	4 (4.82%)					2 (2.41%)	2 (2.41%)
Total	832	782 (94.00%)	50 (6.00%)	6 (0.72%)	5 (0.60%)	3 (0.36%)	2 (0.24%)	21 (2.52%)	13 (1.56%)

### Abnormal NIPT results

A total of 573 patients with abnormal NIPT results, including chromosomal aneuploidy and CNVs detected by NIPT, were evaluated using CMA. Among these patients, a total of 224 patients (224/573, 39.09%) had fetuses with chromosomal abnormalities, including 150 (150/573, 26.18%) with aneuploidy, 7 (7/573, 1.22%) with ROH, and 67 (67/573, 11.69%) with CNVs. The 573 patients with abnormal NIPT results were categorized into 5 subgroups according to distinct chromosomal abnormalities. In the abnormal chr21 group, among the 23 patients, 16 had fetuses with trisomy 21, while one had a fetus with a CNV (VUS) (see Patient 40 in Schedule 2). In the abnormal chr18 group, among the 22 patients, 3 had fetuses with trisomy 18, and 8 had fetuses with CNVs (P/LP) (see Patients 115, 116, 118, and 120–124 in Schedule 1). In the abnormal chr13 group, among the 12 patients, 3 had fetuses with trisomy 13, and 3 patients had fetuses with CNVs. Among the 3 patients whose fetuses had CNVs in the abnormal chr13 group, 2 were identified with CNVs (P/LP) (see Patients 57–58 in Schedule 1) and one was identified with CNV (VUS) (see Patient 27 in Schedule 2). Among 235 patients whose fetuses had other autosomal abnormalities, chromosomal abnormalities were detected in 54 (54/235, 23.0%) fetuses, including 6 (6/235, 2.55%) with aneuploidy, 6 (6/235, 2.55%)with ROH, 42 (42/235, 17.87%) with CNVs. Among 281 patients whose fetuses had sex chromosomal abnormalities, 122 fetuses had sex chromosome aneuploidy, 1 had ROH, and 13 had CNVs (see Table 4). Moreover, the detection rate of abnormal chr21 was significantly higher than that of other autosomal abnormalities (73.91% vs. 22.98%, p < 0.001). The detection rate of sex chromosomal abnormalities was also significantly higher than that of other autosomal abnormalities (48.04% vs. 22.98%, p < 0.001).

**Table 4:** CMA results of 573 samples with abnormal NIPT.

Subgroups	Total	Normal	Abnormal	Aneuploidy	ROH	CNVs (P/LP)	CNVs (VUS)
Abnormal chr21	23 (4.01%)	6 (26.09%)	17 (73.91%)	16 (69.57%)			1 (4.35%)
Abnormal chr18	22 (3.84%)	11 (50.00%)	11 (50.00%)	3 (13.64%)		8 (36.36%)	
Abnormal chr13	12 (2.09%)	6 (50.00%)	6 (50.00%)	3 (25.00%)		2 (16.67%)	1 (8.33%)
Other autosomes	235 (41.01%)	181 (77.02%)	54 (22.98%)	6 (2.55%)	6 (2.55%)	28 (11.91%)	14 (5.96%)
Sex chromosomes	281 (49.04%)	145 (51.06%)	136 (48.04%)	122 (43.42%)	1 (0.36%)	11 (3.91%)	2 (0.71%)
Total	573	349 (60.91%)	224 (39.09%)	150 (26.18%)	7 (1.22%)	49 (8.55%)	18 (3.14%)

### Abnormal ultrasound results

A total of 1379 patients showed fetal anomalies on ultrasound scan, CMA detected chromosome abnormalities in 127 cases (127/1379, 9.21%), including 61 fetuses with chromosomal aneuploidy (61/1379, 4.42%) and 66 fetuses with CNVs (66/1379, 4.79%). According to the characteristics of ultrasound, a total of 1379 patients with ultrasound abnormalities were categorized into five subgroups: the multiple (two or more than two) structural abnormalities subgroup with 27 patients (27/1379, 1.96%), the single-system structural abnormality subgroup included 447 patients (477/1379, 32.41%), the single ultrasound soft marker abnormalities subgroups included 774 patients (56.13%, 774/1379), the multiple (two or more than two) ultrasound soft marker abnormalities subgroups included 70 patients (70/1379, 5.08%), and the structural abnormalities combined with soft marker abnormalities subgroup included 61 patients (61/1379, 4.42%). Among the five subgroups, the chromosomal abnormality rate was the highest in the group with multiple structural abnormalities (18.52%, 5/27), while it was lowest in the group with a single ultrasound soft marker abnormality (75/779, 9.69%). In the subgroup of single-system structural abnormalities, cardiovascular system abnormalities were the most common (175/447, 39.15%), with chromosomal abnormalities detected by CMA in 5.71% (10/175) of patients. The second most common category was genitourinary system abnormalities (91/447, 20.35%), where CMA identified chromosomal abnormalities in 5.49% (5/91) of patients. The prevalence of chromosomal abnormalities among patients with musculoskeletal system abnormalities is the highest (19.05%, 4/21), followed by that among patients with central nervous system abnormalities (15.15%, 5/33). In the single ultrasound soft marker abnormality subgroup, thickened nuchal translucency (29.07%, 225/774) was the most prevalent abnormality, with chromosome abnormalities accounting for 18.22% (41/225), followed by choroid plexus cyst (16.41%, 127/774), with chromosome abnormalities accounting for 7.09% (9/127). However, the detection rate of chromosomal abnormalities with renal echogenicity enhancement was the highest (28.57%, 2/7), followed by that of nuchal cystic hygroma (22.22%, 8/36) (see Table 5).

**Table 5:** CMA results of 1379 samples with ultrasound abnormalities.

Subgroups	Total	Normal	Abnormal	Common autosomal aneuploidies			Sex chromosome aneuploidy	Rare autosomal aneuploidy	CNVs (P/LP)	CNVs (VUS)
				T21	T18	T13				
Abnormality of a single ultrasonic soft marker	774 (56.13%)	699 (90.31%)	75 (9.69%)	26 (3.36%)	10 (1.29%)	1 (0.13%)	5 (0.65%)	1 (0.13%)	24 (3.10%)	8 (1.03%)
Thickened nuchal translucency	225 (16.32%)	184 (81.78%)	41 (18.22%)	16 (7.11%)	6 (2.67%)	1 (0.44%)	2 (0.89%)		12 (5.93%)	4 (1.78%)
Aplasia/hypoplasia of the nasal bone	29 (2.10%)	28 (96.55%)	1 (3.45%)						1 (3.45%)	
Intracardiac echogenic focus	48 (3.48%)	48 (100%)								
Persistent left superior vena cava	12 (0.87%)	12 (100%)								
Ventriculomegaly	104 (7.54%)	97 (93.27%)	7 (6.73%)	4 (3.85%)					2 (1.92%)	1 (0.96%)
Single umbilical artery	29 (2.10%)	27 (93.10%)	2 (6.90%)					1 (3.45%)	1 (3.45%)	
Choroid plexus cyst	127 (9.21%)	118 (92.91%)	9 (7.09%)	2 (1.57%)	3 (2.36%)				3 (2.36%)	1 (0.79%)
Echogenic bowel	45 (3.26%)	44 (97.78%)	1 (2.22%)						1 (2.22%)	
Enlarged posterior fossa	14 (1.02%)	14 (100%)								
Renal echogenicity enhancement	7 (0.51%)	5 (71.43%)	2 (28.57%)						2 (28.57%)	
Polyhydramnios	38 (2.76%)	37 (97.37%)	1 (2.63%)						1 (2.67%)	
Oligohydramnios	10 (0.73%)	9 (90.00%)	1 (10.00%)						1 (10%)	
Nuchal cystic hygroma	36 (2.61%)	28 (77.78%)	8 (22.22%)	4 (11.11%)	1 (2.78%)		3 (8.33%)			
Aberrant subclavian artery	50 (3.63%)	48 (96.00%)	2 (4.00%)							2 (4.00%)
Abnormality of multiple ultrasonic soft markers	70 (5.08%)	63 (90.00%)	7 (10.00%)	1 (1.43%)			2 (2.86%)		3 (4.29%)	1 (1.43%)
Structural anomaly in a single system	447 (32.41%)	417 (93.29%)	30 (6.71%)	4 (0.90%)	2 (0.45%)		1 (0.22%)		15 (3.35%)	8 (1.79%)
Cardiovascular system	175 (12.69%)	165 (94.29%)	10 (5.71%)	2 (1.14%)					6 (3.43%)	2 (1.14%)
Musculoskeletal system	21 (1.52%)	17 (80.95%)	4 (19.05%)				1 (4.76%)		2 (9.52%)	1 (4.76%)
Pleural abnormalities	46 (3.34%)	46 (100%)								
Genitourinary system	91 (6.60%)	86 (94.51%)	5 (5.49%)						2 (2.20%)	3 (3.30%)
Abdominal abnormalities	49 (3.55%)	43 (87.76%)	6 (12.24%)	2 (4.08%)	1 (2.04%)				2 (4.08%)	1 (2.04%)
Faciocervical system	13 (0.94%)	13 (100%)								
Vascular malformations	7 (0.51%)	7 (100%)								
Central nervous system	33 (2.39%)	28 (84.85%)	5 (15.15%)		1 (3.03%)				3 (9.09%)	1 (3.03%)
Sacrococcygeal teratoma	12 (0.87%)	12 (100%)								
Structural anomaly in multiple systems	27 (1.96%)	22 (81.48%)	5 (18.52%)	1 (3.70%)	2 (7.41%)				2 (7.41%)	
Structural anomaly combined with soft marker abnormalities	61 (4.42%)	51 (83.61%)	10 (16.39%)	2 (3.28%)	3 (4.92%)				5 (8.20%)	
Total	1379	1252 (90.79%)	127 (9.21%)	34 (2.47%)	17 (1.23%)	1 (0.07%)	8 (0.58%)	1 (0.07%)	49 (3.55%)	17 (1.24%)

### Other indications

Other indications for prenatal examination include intrauterine growth restriction, history of adverse pregnancy outcomes, maternal request, in vitro fertilization, drug use or exposure to toxic substances during pregnancy, consanguineous marriage, and parental anxiety. Among 960 patients with other indications, CMA verified chromosomal aneuploidy in the fetuses of 8 patients (8/960, 0.83%) and CNVs in the fetuses of 41 patients (41/960, 4.27%) (see Table 1). Among 960 patients with other indications, 633 had a history of adverse pregnancy outcomes, and CMA identified 6 patients whose fetuses had chromosome aneuploidy (0.95%, 6/633) and 22 patients whose fetuses had CNVs (6.47%, 22/633). In this group, the highest proportion of CNVs (7/120, 5.83%) was detected in 120 fetuses with intrauterine growth restriction, with 5 cases of CNVs (P/LP) and 2 cases of CNVs (VUS). Five fetuses with CNVs (P/LP) included 1 case with a 5q23.2q35.3 duplication with an Xq23q28 deletion, 1 case with 16p11.2 deletion syndrome, 2 cases with Wolf-Hirschhorn syndrome and 1 case with a 13q31.1q32.1 deletion.

### Detection of ROH by the CMA platform

CMA was used to identify 11 fetuses with ROH (see Schedule 2), including 1 with a whole X chromosome, 1 with a whole chromosome 4, 2 with a long arm fragment on chromosome 15, 1 with a short arm fragment on chromosome 16, 2 with a partial fragment on chromosome 8, 1 with a short arm fragment on chromosome 5, 1 with a long arm fragment on chromosome 12 and 8, 1 with a long arm fragment on chromosome 10, and 1 case with a long fragment on chromosome 2, 18 and 13.

### Pregnancy follow-up outcome

A total of 4211 pregnant women were followed up (see Table 6). Among the 3731 fetuses with normal CMA results, 3568 fetuses (3568/3731, 95.63%) were confirmed to be normal after birth, while 100 pregnant women (100/3731, 2.68%) underwent pregnancy termination for various reasons, Fetal abnormalities were observed postnatally in 45 fetuses (45/3731, 1.21%). Among 100 women who terminated their pregnancy, 56 fetuses were induced by abnormal ultrasound structure, 11 fetuses were fetal death, 11 fetuses were accidental abortion, 4 fetuses were induced by monogenic disease, 5 fetuses were mosaicism determined by karyotype, 4 fetuses were placental abruption, 9 fetuses declined labor induction for personal reasons. Among the 45 fetuses with postnatal abnormalities, the most prevalent anomaly observed was cardiac structural abnormalities (15/45, 33.33%), followed by postnatal mortality (5/45, 11.11%). Among the 404 fetuses with aneuploidies or CNVs (P/LP), 332 pregnant women (332/404, 82.18%) underwent pregnancy termination, and 70 (70/404, 17.33%) continued their pregnancy. Among the 70 instances of childbirth, 65 fetuses (65/404, 16.09%) were confirmed to be normal, and 5 (5/404, 1.24%) fetuses exhibited postnatal abnormalities. These 5 abnormalities included 1 case of postnatal death caused by trisomy 2 mosaicism, 3 cases of ichthyosis, and 1 case of atrial septal defect and ventricular septal defect. Among the 76 pregnant women whose fetuses with CNVs (VUS), 30 (30/76, 39.47%) opted for pregnancy termination, while 45 (45/76, 59.21%) opted to continue their pregnancy. Among the 45 instances of childbirth, 44 (44/76, 57.89%) fetuses were born without abnormalities, and one (1/76, 1.32%) fetus exhibited postnatal abnormal development (six fingers in the right hand). A total of 21 pregnant women declined to provide information, comprising 18 fetuses with normal CMA results, 2 fetuses with CNVs (P/LP) and 1 fetus with CNVs (VUS). The postnatal follow-up of the fetuses revealed that the proportions of abnormal and normal fetuses with aneuploidies or CNVs (P/LP) were 7.14% (5/70) and 92.86% (65/70), respectively. Furthermore, among the fetuses with CNVs (VUS), the percentages were 2.22% (1/45) and 97.78% (44/45), respectively.

**Table 6:** Follow-up results of 4211 pregnant women tested for CMA.

CMA results	Total	Normal child after birth	Induced delivery	Abnormal child after birth	Refuse
Normal	3731 (88.60%)	3568 (95.63%)	100 (2.68%)	45 (1.21%)	18 (0.48%)
Aneuploidies or CNVs (P/LP)	404 (9.59%)	65 (16.09%)	332 (82.18%)	5 (1.24%)	2 (0.49%)
CNVs (VUS)	76 (1.81%)	44 (57.89%)	30 (39.47%)	1 (1.32%)	1 (1.32%)
Total	4211	3677 (87.32%)	462 (10.97%)	51 (1.21%)	21 (0.50%)

## Discussion

In this study, we investigated the clinical significance of CMA technology in prenatal diagnosis among a cohort of 4211 pregnant women. The overall abnormal detection rate of CMA was 11.4%, with 5.82% for chromosomal numerical abnormalities and 5.58% for copy number variations (CNVs). The detection rate of the group with abnormal NIPT results was the highest (39.1%, 224 /573), followed by the group with the abnormal ultrasound (9.2%, 127/1379), while the group with other indications had the lowest detection rate (5.2%, 50/960). We compared the detection rate of CNVs (P/LP) detected by CMA in individuals with various indications for prenatal diagnosis, as reported in the literature. The overall detection rate of CNVs (P/LP) ranged from 1.4 to 8.3%^16–21^, and the findings of our study (3.78%) fell within this range. In the AMA-only group, the abnormal MSS results group, and the abnormal NIPT results group, the detection rates of CNVs (P/LP) by CMA were 2.35%, 2.52%, and 8.55%, respectively. These results were consistent with previously reported detection rates in the literature ranging from 0.84% to 5.8%, 1.6% to 8.0%, and 0.76% to 35.3%, respectively^16–22^. However, in the group with abnormal ultrasound results, the detection rate of CNVs (P/LP) by CMA was 3.55%, which was lower than that reported in previous studies (4.5–7.9%)^16,17,19,21^, particularly within the subgroup of single-system structural abnormalities (3.35% vs. 3.66–10.9%)^17,21,22^ and multiple system structural abnormalities (7.41% vs. 8.57–22.6%)^17,21,22^. The discrepancy between the previous studies and this study may be attributed to factors such as patient selection, potential biases, the availability of reimbursed tests, or the utilization of conventional karyotyping methods for excluding macroscopically visible fetal chromosomal aberrations.

In the group with abnormal NIPT results, chromosome aneuploidy and CNVs were detected across all 24 chromosomes through NIPT. Due to the use of different NIPT platforms, which employ varying sequence read depths and algorithms provided by multiple providers, accurate assessment of positive predictive values was not feasible. The positive predictive values for the detection of other autosomes were consistently lower than those observed for abnormal 21, abnormal 18, abnormal 13, and sex chromosomes in the general population, irrespective of the platform employed. Therefore, the screening efficacy of NIPT for detecting other chromosome aneuploidies and CNVs remains limited. Due to the technical limitations of NIPT, the detected material is derived from placental cell-free DNA, leading to false-positive results. Among the 573 patients with abnormal NIPT results in this study, 349 had normal results after prenatal diagnosis. Possible factors included confined placental mosaicism, early disappearance or cessation of the development of one of the twins, and maternal chromosomal abnormalities or maternal diseases.

Ultrasound soft markers are frequently observed in fetuses with chromosomal abnormalities, however, the indications to perform CMA in fetuses with soft markers are not standardized. Several typical cases were identified in this study. Two fetuses with 6p25 deletion syndrome were detected, and all clinical indications suggested thickened nuchal translucency. Three fetuses with fetal renal cysts and diabetes syndrome showed enhanced renal echo during prenatal ultrasound examination. These findings are consistent with the published literature showing that the most common pathogenic CNV in fetuses with renal and urinary tract abnormalities is 17q12 microdeletion, which leads to renal cysts and diabetic syndrome^23^. The presence of clinically significant CNVs may be observed when prenatal ultrasound indicates thickened nuchal translucency or enhanced renal echo. Fetuses with ultrasound soft marker abnormalities exhibited varying incidence rates of CNVs (P/LP). Our research revealed a small but not statistically significant increase in the likelihood of clinically relevant CNVs in fetuses with one or more ultrasound soft markers (3.1% vs. 4.29%, p = 0.853), consistent with previous investigations^24^. These findings suggested that CMA should be performed in pregnant women when two or more ultrasound soft markers are detected by ultrasound.

Among the subgroup of fetuses with ultrasound structural abnormalities, the detection rate of CNVs (P/LP) was the highest in the group of fetuses with structural anomalies combined with soft marker abnormalities (8.2%), followed by those with multiple system structural abnormalities (7.41%). The detection rate of CNVs (P/LP) in fetuses with a single-system malformation is similar to that previously reported (3.1–7.9%)^25^, with skeletal (9.52%), genitourinary (2.2%), central nervous (9.09%) and cardiovascular system abnormalities (3.43%) being the most commonly associated with CNVs (P/LP). These results showed that CMA should be recommended when fetal ultrasound reveals multiple structural abnormalities, especially in skeletal, genitourinary, central nervous, and cardiovascular system abnormalities. In this study, Two fetuses with Emanuel syndrome were identified, and CMA results showed 11q23.3q25 region duplication and 22q11.1q11.21 region duplication. Prenatal ultrasound revealed a single umbilical cord and underdeveloped cerebellar vermis in one fetus, while the other exhibited a posterior fossa anomaly. These are the most common defects of ES and might be diagnosed in early pregnancy^26^. The clinical phenotype of the 22q11.2 proximal deletion exhibits significant heterogeneity, primarily including heart defects, palatal abnormalities, developmental retardation, and immunodeficiency. In this study, Two fetuses with a 22q11.2 proximal deletion had cardiac defects: one exhibited right foot varus and left ventricular punctate strong echoes, and the other presented with a right aortic arch. 22q11.2 deletion is frequently observed in fetuses with heart defects diagnosed prenatally^27^. Therefore, CMA can be used as a detection method for prenatal echocardiography abnormalities.

The phenotype resulting from a susceptibility CNV is unpredictable due to incomplete penetrance and variable expressivity^28,29^. These features have not been systematically described although the clinical phenotypic features associated with postnatal recurrent microdeletion/duplication syndromes are well-defined, these features have not been systematically described in prenatal cases due to the limitations of prenatal identification. In our study, the most common recurrent microdeletion/duplication syndromes were 1q21.1 deletion syndrome (10 fetuses) and 15q11.2 deletion syndrome (10 fetuses). 22q11.2 duplication syndrome (9 fetuses), 16p13.11 duplication syndrome (9 fetuses), proximal 16p12.2 microdeletion syndrome (7 fetuses), 16p11.2 microdeletion syndrome (4 fetuses), and 16p11.2 duplication syndrome (3 fetuses), 16p13.11 deletion syndrome (2 fetuses) were also common in this study. Among these fetuses, only 7 (7/54, 12.2%) exhibited structural abnormalities and all pregnancies were terminated. Moreover, we observed that the proportion of normal fetuses after birth is greater in recurrent microdeletion/duplication syndromes with penetrance of less than 10%. For example, approximately 90% of fetuses with 15q11.2 deletion syndrome and 16p13.11 duplication syndrome have a normal outcome. Therefore, we recommend not informing couples about CNVs classified as loci with penetrance of less than 10%. These potential neurodevelopmental sites may have some structural abnormalities and may not have obvious clinical indications during pregnancy, which will increase parents' anxiety about fetal development. The precise ultimate phenotype remains unknown, posing significant challenges for genetic counseling.

The single nucleotide polymorphism array (SNP array) technology of CMA can detect not only CNVs, but also ROH, uniparental disomy (UPD), and low-level mosaics^30^.As chromosomes 6,7,11,14,15 and 20 are known to be associated with parental-specific expression genes, further testing is necessary to clarify the diagnosis and distinguish between ROH and UPD. In clinical practice, child-parent trio analysis through CMA is often required^31^. This study reported two cases of fragmented ROH involving the long arm of chromosome 15. Due to AMA and the absence of future childbearing plans, both couples abandoned further diagnostic tests and terminated their pregnancies, so we could not determine the pathogenicity of fragmented ROH involving the long arm of chromosome 15. A case of fragmented ROH on the short arm of chromosome 5 was reported. The karyotype of this fetus was 46, XY, del(5)(p13)[32]/46, XY[70], which led to mosaic loss in the critical region causing Cri du chat syndrome. Consequently, the couple decided to induce labor. When a critical region causing microdeletion syndrome exhibits ROH, it is imperative to complement this region with karyotyping analysis to ascertain its pathogenicity. The presence of multiple large regions of ROH on various chromosomes may indicate a potential consanguineous relationship between the tested individual's parents, either close or distant^32^. In this study, one fetus was found to have multiple large regions of ROH on various chromosomes due to consanguineous marriage between the parents. The couple ultimately decided to terminate the pregnancy. The presence of ROH on the whole of chromosome 4 was identified in our study. In the advanced stages of gestation, intrauterine growth restriction led to fetal demise within the uterus. This finding was consistent with previous studies that fetuses with ROH frequently exhibited the most prevalent prenatal manifestation of intrauterine growth restriction^33^. For 6 fetuses involving other chromosome fragmentary ROH regions, all six couples opted to continue their pregnancies, and subsequent assessments confirmed normal fetal development postpartum. Due to the complex pathogenesis of ROH, encompassing gene imprinting effects, homozygous recessive gene mutations, and low-level chromosomal mosaics, a comprehensive assessment of prognosis should be conducted by combining ultrasound findings, family verification, whole exome sequencing, and other relevant factors.

The pregnancy outcomes of all women were evaluated in this study. Although the majority of women with fetuses diagnosed with aneuploidy or CNV(P/LP) opted for pregnancy termination, 70 women whose fetuses with aneuploidy or CNVs (P/LP) decided to continue their pregnancy, and subsequent follow-up revealed that 65 of the newborns exhibited normal phenotypes. Notably, among these patients, 50% had fetuses with sex chromosome aneuploidy. The results of our investigation indicated that an increasing number of people are accepting of children with sex chromosome aneuploidy. In this study, 76 fetuses with CNVs (VUS) were found, for a detection rate of 1.85%. After follow-up, a total of 45 fetuses were delivered, and among these fetuses, 44 exhibited normal postnatal outcomes. This finding indicates that fetuses with CNVs (VUS) are most likely to have a good pregnancy outcome. Furthermore, reporting VUS in prenatal diagnosis may present challenges for genetic counseling, place pressure on pregnant women and their families, and lead to excessive termination of pregnancy. Therefore, in subsequent stages, further case accumulation and long-term follow-up are needed to comprehensively evaluate the prognosis. This study has significant implications for the development of future genetic counseling guidelines.

Due to the limited availability of detailed genotype–phenotype information for some fetuses, this retrospective study is limited in terms of data acquisition. For instance, some pregnant women have poor compliance or refuse to undergo testing due to financial constraints, while others may experience information loss during the referral process, resulting in the potential omission of high-risk indications for certain fetuses and ultimately introducing bias into the study findings. Furthermore, incomplete clinical examinations in fetuses with a young gestational age may lead to insufficient descriptions of certain clinical phenotypes. Ultimately, the absence of parental data presents a significant challenge in assessing the pathogenicity of certain CNVs, thereby complicating our clinical genetic counseling efforts.

The CMA test is applicable for all prenatal clinical indications because it enhances the detection of clinically significant CNVs. Clinicians should duly apprise pregnant women about the potential risk of undetectable CNVs through biochemical screening or most existing NIPT platforms, and emphasize the value of CMA in prenatal diagnosis for informed decision-making.

## Materials and methods

### Patients and clinical indications

All patients who underwent invasive prenatal diagnosis by CMA at Linyi Women and Children's Hospital between 2016 and 2022 in Shandong, China. Clinical samples (chorionic villi and amniotic fluid) were obtained by ultrasound-guided abdominal chorionic villus sampling (CVS) and amniocentesis. After receiving detailed genetic counseling before testing, each participant signed a written informed consent form. This study was approved by the Ethics Committee of Linyi Maternal and Child Hospital (No. KYL-YXLL-2022017). The research was conducted in accordance with the relevant guidelines and clinical norms.

### CMA analysis

Fetal uncultured genomic DNA was extracted by a DNA extraction kit (TIANamp Micro DNA Kit, China). The whole genome CytoScan 750 K array (Thermo Fisher Scientific, USA) was used for CMA according to the manufacturer's instructions. The original data were analyzed by chromosome analysis software 4.2 (Thermo Fisher Scientific, USA) of genome version GRCh37/hg19. The results were classified according to genetic mode (familial or de novo), CNV length, genes involved, their classification, and literature information. The online databases consulted included PubMed (http://www.ncbi.nlm.nih.gov/PubMed), Decipher (https://www.deciphergenomics.org/), UCSC (https://genome.ucsc.edu/index.html), ClinGen (https://search.clinicalgenome.org/), Clinvar (https://www.ncbi.nlm.nih.gov/clinvar/), HGMD (https://www.hgmd.cf.ac.uk/ac/index.php) and DGV (http://dgv.tcag.ca/dgv/app/home). The results were classified as pathogenic (P), likely pathogenic (LP), variant of uncertain significance (VUS) and normal (including likely benign and benign) according to the 2019 ACMG guidelines. Benign (common polymorphism) and/or likely benign CNVs were detected, and the reported results were normal. Regions of homozygosity (ROH) involving chr 6, chr 7, chr 11, chr 14, chr 15, chr 20 with 5 Mb (at the end of the chromosome) or 10 Mb (not at the end of the chromosome) on one of the chromosomes were also reported, while no ROH was detected on the other chromosomes^14,15^.

### Statistical analysis

SPSS 21.0 (Chicago, USA) was used for the statistical analysis. Classified variables are expressed as numbers and percentages, and the chi-square test was used for comparisons. A two-sided p-value < 0.05 was considered statistically significant. Linear regression models with gamma values were used to assess the changes in abnormal rates in different age groups.

### Supplementary Information

Supplementary Information.

## Data Availability

The data that support the findings of this study are available from the corresponding author upon reasonable request.
