# Supplementary material for: Chromosomal abnormalities detected by chromosomal microarray analysis and pregnancy outcomes of 4211 fetuses with high-risk prenatal indications
Source: Sci Rep. 2024 Jul 10;14:15920. doi: 10.1038/s41598-024-67123-5 (PMC11237145; doi:10.1038/s41598-024-67123-5)
Supplement: Supplementary file 1 — Supplementary Information. [file 41598_2024_67123_MOESM1_ESM.pdf]

Chromosomal abnormalities detected by chromosomal microarray analysis and pregnancy outcomes of 4211 fetuses with high-risk prenatal indications

Huafeng Li, Juan Hu, Qingyu Wu, Jigang Qiu, Li Zhang, Jinping Zhu*

Schedule 1: Pathologic, Likely pathologic copy number variations found in fetuses with indication for genetic study by CMA

Case	Age	Clinical indications	CMA Results[hg19]	Size (Mb)	Disorders	Interpretation	Outcome
1	26	PLSVC	arr[hg19] 1q21.1q21.2(146,023,922-147,885,600)x1	1.8	1q21.1deletion syndrome	P	TOP
2	33	NIPT(+)	arr[hg19] 1q21.1q21.2(145,895,746-147,830,830)x1	1.9	1q21.1deletion syndrome	P	TOP
3	25	NIPT(+)	arr[hg19]1q21.1q21.2(146,023,922-147,929,115)x1	1.9	1q21.1deletion syndrome	P	live born
4	31	High risk maternal serum screening	arr[hg19] 1q21.1q21.2(145,895,747-147,830,830)x1	1.93	1q21.1 deletion syndrome	P	TOP
5	32	High risk maternal serum screening	arr[hg19] 1q21.1q21.2(145,895,747-147,830,830)x1	1.93	1q21.1 deletion syndrome	P	TOP
6	36	Adverse pregnancy,AMA	arr[hg19]1q21.1q21.2(146,106,723-147,830,830)x1	1.7	1q21.1deletion syndrome	P	TOP
7	31	Microcephaly	arr[hg19]1q21.1q21.2(146,023,922-147,830,830)x1	1.8	1q21.1 deletion syndrome	P	TOP

8	34	Mental retardation of pregnant women	arr[hg19]1q21.1q21.2(146,023,922-147,830,830)x1	1.8	1q21.1 deletion syndrome	P	TOP
9	32	Adverse pregnancy	arr[hg19]1q21.1q21.2(146,106,724-147,830,830)x1	1.7	1q21.1 deletion syndrome	P	live born
10	32	Adverse pregnancy	arr[hg19]1q21.1q21.2(146,106,724-147,830,830)x1	1.9	1q21.1 deletion syndrome	P	TOP
11	25	NIPT(+)	arr[hg19] 1q43q44(237,234,000-249,224,684)x1 arr[hg19] 18p11.32p11.21(136,228-13,971,993)x3	12.0/13.8	1q43-q44 deletion syndrome/ 18p11.32p11.21 duplication	P	TOP
12	23	ventriculomegaly	arr[hg19] 1p32.1p31.3(60,975,516-61,980,993)x1	1.0	1p32-p31deletion syndrome	P	TOP
13	23	SUA,Renal echogenicity enhancement	arr[hg19] 1q42.11q44(224,225,265-248,587,574)x3	24.3	Megalencephaly-Polymicrogyria-Polydactyly-Hydrocephalus syndrome 2	P	TOP
14	25	EB	arr[hg19]2q11.1q12.2(95,372,685-106,637,549)x3	11.3	2q11.2 duplication syndrome	P	TOP
15	30	Adverse pregnancy	arr[hg19] 3q29(195,718,751-197,340,833)x1	1.6	3q29 deletion syndrome	P	TOP
16	31	NT(+)	arr[hg19] 3q29(195,678,474-197,403,146)x1	1.7	3q29 deletion syndrome	P	TOP

17	28	Cerebellar vermis hypoplasia， Third ventricle dilatation	arr[hg19] 4p16.3p15.2(68,345-23,931,209)x1	23.8	Wolf-Hirschhorn syndrome	P	TOP
18	32	IUGR	arr[hg19] 4p16.3(68,346-3,536,176)x1<br>arr[hg19] 17q25.3(79,300,174-81,041,823)x3	3.46/1.74	Wolf-Hirschhorn syndrome	P	TOP
19	29	IUGR	arr[hg19] 4p16.3p15.2(68,346-24,703,885)x1	24.6	Wolf-Hirschhorn syndrome	P	TOP
20	23	NIPT(+)	arr[hg19] 4p16.3p15.2(68,345-23,994,155)x1<br>arr[hg19] 4q34.3q35.2(179,642,221-190,806,055)x1<br>arr[hg19] 4p15.2(23,996,554-25,268,505)x3	11.1/1.3	Wolf-Hirschhorn syndrome	P	TOP
21	29	Hydrops fetalis	arr[hg19] 4q34.1q35.2(173,929,353-190,957,460)x3<br>arr[hg19] 16p13.12p13.11(14,769,089-16,458,424)x1	17/1.7	4q34.1q35.2 duplication/16p13.11 deletion syndrome	P	TOP
22	30	Renal dysplasia	arr[hg19]4q28.1q35.2(123,940,073-190,957,460)x3<br>arr[hg19]21q22.2q22.3(41,812,092-48,093,361)x1	67/6.28	Auriculo-acro-renal syndrome/21q22.2q22.3 deletion	P	TOP
23	22	VSD,Duodenal atresia	arr[hg19]4q28.1q31.1(127,877,698-140,761,997)x1	12.9	4q28.1q31.1 deletion	P	TOP
24	27	High risk maternal serum screening	arr[hg19] 5p15.33p13.3(113,576-33,171,259)x1	33.0	Cridu Chat Syndrome	P	TOP
25	28	NIPT(+)	arr[hg19] 5p15.33p15.1(113,576-17,373,382)x1<br>arr[hg19] 9p24.3p22.3(208,454-14,837,973)x3	17.3/14.6	Cridu Chat Syndrome/Coffin-Siris syndrome	P	TOP

26	33	Adverse pregnancy	arr[hg19] 5p15.33p15.2(113,576-14,075,834)x1 arr[hg19] 6p25.3p22.3(381,117-19,062,056)x3	14/18.7	cridu chat syndrome	P	TOP
27	24	Mental retardation of pregnant women	arr[hg19] 5p15.33p15.2(113,576-11,118,144)x1	11.0	cridu chat syndrome	P	TOP
28	33	High risk maternal serum screening	arr[hg19] 5p15.33p15.31(113,577-6,844,078)x1	6.7	cridu chat syndrome	P	TOP
29	37	NIPT(+),AMA	arr[hg19]5q21.3q22.2(108,594,395-112,707,610)x1	4.1	familial adenomatous polyposis-1	P	TOP
30	20	NT(+)	arr[hg19] 6p25.3(156,974-2,117,006)x1	2.0	6p25.3 deletion syndrome	P	TOP
31	43	NT(+),AMA	arr[hg19] 6p25.3p25.2(156,974-3,887,339)x1	3.7	6p25.3 deletion syndrome	P	TOP
32	26	High risk maternal serum screening	arr[hg19]6q16.3q22.31(104,149,016-119,867,071)x1	15.7	Interstitial 6q deletion syndrome	P	TOP
33	27	Requirements for pregnant women	arr[hg19] 7q11.23(72,701,098-74,154,404)x1	1.5	Williams-Beuren Syndrome	P	TOP
34	27	High risk maternal serum screening	arr[hg19] 7q11.23(72,550,101-74,374,748)x1	1.8	Williams-Beuren Syndrome	P	TOP

35	31	Enhanced renal echo	arr[hg19] 7q31.33q35(129600564-146622078)x3	17.0	7q31.3q35 duplication	P	TOP
36	34	NT(+)	arr[hg19] 7q36.3(155,347,675-158,404,870)x4	3.1	Triphalangeal thumb-polysyndactyly syndrome	P	TOP
37	37	Adverse pregnancy,AMA	arr[hg19]7q36.1q36.3(151,262,062-159,119,707)x1 arr[hg19]7q35q36.1(144,652,788-151,252,408)x3	7.8/6.6	7q36 deletion syndrome/7q35q36.1duplication	P	TOP
38	39	AMA	arr[hg19] 7p22.3p22.1(43,377-5,016,415)x1 arr[hg19] 11q23.3q25(117,958,764-134,937,416)x3	4.9/16.9	7p22.3p22.1 deletion / 11q23.3q25 duplication	P	TOP
39	35	AMA	arr[hg19] 8p23.3p23.1(158,048-6,950,383)x1	6.8	8p23 deletion syndrome	P	TOP
40	35	AMA	arr[hg19] 8p23.3p23.1(158,048-6,950,383)x1	6.8	8p23 deletion syndrome	P	TOP
41	26	NIPT(+)	arr[hg19] 8p23.3p23.1(158,049-6,928,011)x1	6.8	8p23 deletion syndrome	P	live born
42	43	NIPT(+),AMA	arr[hg19] 8p23.3p23.1(158,049-6,950,383)x1 arr[hg19] 8p23.1p11.21(12,532,774-42,005,196)x3	6.7/29.4	8p inverted duplication deletion syndrome	P	TOP
43	37	NIPT(+),AMA	arr[hg19] 9p24.3p24.1(208,454-4,800,164)x1	4.6	9p24 deletion	P	TOP

44	38	VM(+),AMA	arr[hg19] 9p24.3p21.3(208,454-23,771,501)x3 arr[hg19] 8p23.3(158,048-1,807,316)x1	23.5/1.3	9p24.3p21 duplication/8p23.3 deletion	P	TOP
45	36	AMA	arr[hg19]9q22.1q22.32(90,671,252-98,785,793)x1	8.1	Basal cell nevus syndrome	P	TOP
46	22	NT(+)	arr[hg19] 10p15.3p13(100,048-14,160,295)x1 arr[hg19] 18q21.31q23(55,651,773-78,013,728)x3	14.1/22.4	10p15.3p13 deletion /18q21.31q23 duplication	P	TOP
47	19	NIPT(+)	arr[hg19] 10q11.21q11.23(44,379,291-50,610,474)x1	6.23	10q11.21q11.23 deletion	P	TOP
48	29	CPC	arr[hg19] 10q26.11q26.3(120,836,116-135,426,386)x1-2[0.34]	14.6	10q26.11q26.3 mosaic deletion	P	TOP
49	22	Oligohydramnios	arr[hg19] 11p12q11(41,381,133-54,925,385)x3	13.5	11p12q11duplication	P	TOP
50	29	VM(+)	arr[hg19] 11q23.3q25(116,683,754-134,937,416)x3 arr[hg19] 22q11.1q11.21(16,888,899-20,312,661)x3	18.2/3.4	Emanuel Syndrome	P	TOP
51	27	SUA	arr[hg19] 11q23.3q25(116,683,754-134,937,416)x3 arr[hg19] 22q11.1q11.21(16,888,899-20,312,661)x3	18.3/3.4	Emanuel Syndrome	P	TOP
52	24	High risk maternal serum screening	arr[hg19] 11p15.5(230,681-2,589,263)x3	2.4	Beckwith-Wiedemann syndrome/ Silver-Russell syndrome	P	TOP

53	25	CPC	arr[hg19]12q15q21.33(70,610,506-89,154,627)x 1	18.5	12q15 deletion syndrome	P	TOP
54	40	NIPT(+),AMA	arr[hg19] 13q11q14.2(19,436,286-50,339,507)x3	30.9	13q11q14.2 duplication	LP	TOP
55	27	IUGR	arr[hg19] 13q31.1q32.1(84,458,941-96,088,400)x1	11.6	Feingold syndrome 2	P	TOP
56	24	Duodenal stenosis or atresia	arr[hg19] 13q21.1q22.2(59,494,652-76,104,670)x1	16.6	13q21.1q22.2 deletion	P	TOP
57	26	NIPT(+)	arr[hg19]13q13.2q21.1(34,863,720-56,245,573)x1	21.4	13q14 deletion syndrome	P	TOP
58	34	NIPT(+)	arr[hg19] 13q32.1q34(96,921,313-115,107,733)x1<br>arr[hg19] 9p24.3p22.1(208,455-19,654,468)x3	18.2/19.4	Holoprosencephaly 5	P	TOP
59	30	NIPT(+)	arr[hg19] 15q11.2q13.1(23,290,787-28,659,911)x1	5.3	Prader-Willi Syndrome	P	TOP
60	41	Adverse pregnancy,AMA	arr[hg19] 15q13.2q13.3(30,370,017-32,444,043)x1	2.1	15q13.3 deletion syndrome	P	TOP
61	30	NIPT(+)	arr[hg19]15q11.2q13.1(23,290,787-28,526,905)x3	5.2	15q11q13 duplication syndrome	P	TOP

62	31	High risk maternal serum screening	arr[hg19]15q11.2q13.3(22,770,421-32,931,921)x4	10.2	15q11q13 duplication syndrome	P	TOP
63	31	Adverse pregnancy	arr[hg19]15q11.2q13.1(23,290,788-28,934,990)x3	5.6	15q11q13 duplication syndrome	P	TOP
64	41	NIPT(+),AMA	arr[hg19]15q11.2q13.1(23,290,788-28,560,664)x3	5.3	15q11q13 duplication syndrome	P	TOP
65	27	Adverse pregnancy	arr[hg19] 15q11.2(22,770,422-23,288,350)x1	0.5	15q11.2 deletion syndrome	P	live born
66	34	High risk maternal serum screening	arr[hg19] 15q11.2(22,770,422-23,282,798)x1	0.5	15q11.2 deletion syndrome	P	live born
67	38	AMA	arr[hg19] 15q11.2(22,770,421-23,282,798)x1	0.5	15q11.2 deletion syndrome	P	live born
68	43	AMA	arr[hg19] 15q11.2(22,770,421-23,288,350)x1	0.5	15q11.2 deletion syndrome	P	live born
69	35	AMA	arr[hg19] 15q11.2(22,770,422-23,288,350)x1	0.5	15q11.2 deletion syndrome	P	live born
70	25	NT(+)	arr[hg19] 15q11.2(22,770,422-23,288,350)x1	0.5	15q11.2 deletion syndrome	P	live born

71	35	NT(+),AMA	arr[hg19] 15q11.2(22,770,422-23,277,436)x1	0.5	15q11.2 deletion syndrome	P	live born
72	27	NB（+）	arr[hg19] 15q11.2(22,770,421-23,288,350)x1	0.5	15q11.2 deletion syndrome	P	TOP
73	30	Requirements for pregnant women	arr[hg19] 15q11.2(22,770,421-23,082,237)x1	0.3	15q11.2 deletion syndrome	P	live born
74	27	Requirements for pregnant women	arr[hg19] 15q11.2(22,770,421-23,277,436)x1	0.5	15q11.2 deletion syndrome	P	live born
75	26	NT(+)	arr[hg19] 16p13.3p13.13(360,919-11,846,439)x3	11.0	16p13 duplication syndrome	P	TOP
76	34	NT(+)	arr[hg19] 16p13.11(15,481,747-16,538,596)x1	1.05	16p13.11 deletion syndrome	P	live born
77	35	NIPT(+),AMA	arr[hg19] 16p13.11(14,910,158-16,458,424)x1	1.5	16p13.11 deletion syndrome	P	live born
78	27	NIPT(+)	arr[hg19] 16q11.2q21(46,503,968-64,669,301)x3	18.1	16q11.2q21 duplication	P	TOP
79	27	IUGR	arr[hg19] 16p11.2(28,485,897-30,190,029)x1	1.7	Proximal 16p11.2 deletion syndrome	P	TOP

80	31	left-handed hexadactyly	arr[hg19] 16p11.2(28,485,897-30,190,029)x1	1.7	Proximal 16p11.2 deletion syndrome	P	TOP
81	20	Cervical lymphangioma,Pleural effusion	arr[hg19] 16p11.2(29,580,020-30,190,029)x1	0.6	Proximal 16p11.2 deletion syndrome	P	TOP
82	37	VSD,AMA	arr[hg19] 16p11.2(28,810,324-29,032,280)x1	0.2	Proximal 16p11.2 deletion syndrome	P	TOP
83	32	High risk maternal serum screening	arr[hg19] 16p13.3(85,880-1,971,336)x1	1.9	Alpha-thalassemia/impaired intellectual development syndrome, deletion type	P	live born
84	31	Separation of double renal pelvis collecting duct system	arr[hg19] 16p11.2(28,371,468-30,176,508)x3	1.8	16p11.2 duplication syndrome	P	TOP
85	26	NIPT(+)	arr[hg19] 16p11.2(29,628,662-30,190,029)x3	0.6	16p11.2 duplication syndrome	P	TOP
86	32	High risk maternal serum screening	arr[hg19] 16p11.2(29,567,297-30,190,029)x3	0.6	16p11.2 duplication syndrome	P	TOP
87	29	Adverse pregnancy	arr[hg19] 16p13.11p12.3(15,481,747-18,231,275)x3	2.8	16p13.11 duplication syndrome	LP	live born
88	29	NIPT(+)	arr[hg19] 16p13.11(15,058,820-16,288,872)x3	1.2	16p13.11 duplication syndrome	LP	live born

89	34	Adverse pregnancy	arr[hg19] 16p13.11(14,929,070-16,508,123)x3	1.6	16p13.11 duplication syndrome	LP	live born
90	34	High risk maternal serum screening	arr[hg19] 16p13.11(15,058,820-16,327,887)x3	1.3	16p13.11 duplication syndrome	LP	live born
91	25	NIPT(+)	arr[hg19]16p13.11p12.3(15,325,072-18,242,713)x3	2.9	16p13.11 duplication syndrome	LP	live born
92	23	High risk maternal serum screening	arr[hg19]16p13.11p12.3(15,481,748-18,242,713)x3	2.8	16p13.11 duplication syndrome	LP	live born
93	31	High risk maternal serum screening	arr[hg19] 16p13.11(15,481,748-16,309,046)x3arr[hg19] 16p12.2(21,841,354-22,442,007)x3	0.8	16p13.11 duplication syndrome	LP	live born
94	40	AMA	arr[hg19] 16p13.11(15,058,821-16,508,123)x3	1.5	16p13.11 duplication syndrome	LP	TOP
95	37	Adverse pregnancy,AMA	arr[hg19] 16p13.11p12.3(15,193,982-18,242,713)x3	3.0	16p13.11 duplication syndrome	LP	live born
96	22	Adverse pregnancy	arr[hg19] 16p12.2(21,841,353-22,442,007)x1	0.6	Proximal 16p12.2 deletion syndrome	LP	live born
97	32	High risk maternal serum screening	arr[hg19] 16p12.2(21,841,353-22,442,007)x1	0.6	Proximal 16p12.2 deletion syndrome	LP	TOP

98	32	SUA	arr[hg19] 16p12.2(21,841,353-22,442,007)x1	0.6	Proximal 16p12.2 deletion syndrome	LP	TOP
99	30	High risk maternal serum screening	arr[hg19] 16p12.2(21,740,199-22,441,961)x1	0.7	Proximal 16p12.2 deletion syndrome	LP	TOP
100	21	Single umbilical artery	arr[hg19] 16p12.2(21,801,889-22,431,031)x1	0.6	Proximal 16p12.2 deletion syndrome	LP	live born
101	42	AMA	arr[hg19] 16p12.2(21,740,200-22,442,007)x1	0.72	Proximal 16p12.2 deletion syndrome	LP	live born
102	21	CPC	arr[hg19] 16p12.2(21,841,353-22,442,007)x1	0.6	Proximal 16p12.2 deletion syndrome	LP	live born
103	18	Enhanced renal echo	arr[hg19] 17q12(34,822,465-36,387,880)x1	1.56	Renal cysts and diabetes syndrome	P	TOP
104	36	Hyperechogenic Kidney,AMA	arr[hg19] 17q12(34,822,465-36,300,466)x1	1.47	Renal cysts and diabetes syndrome	P	TOP
105	32	EB，Hyperechogenic Kidney	arr[hg19] 17q12(34,822,466-36,418,529)x1	1.59	Renal cysts and diabetes syndrome	P	TOP
106	25	High risk maternal serum screening	arr[hg19] 17q12(34,822,466-36,404,555)x1 arr[hg19] Xp22.31(6,455,152-8,135,568)x0	1.58/1.68	Renal cysts and diabetes syndrome/X-linked Ichthyosis	P	TOP

107	38	IUGR,AMA,Oligohydramnios	arr[hg19] 17q12(34,822,466-36,378,678)x3	1.55	17q12 duplication syndrome	P	TOP
108	31	Polyhydramnios	arr[hg19] 17p13.3(525-3,128,009)x1	3.12	Miller-Dieker lissencephaly syndrome	P	TOP
109	23	Mental retardation of pregnant women	arr[hg19] 17p11.2(16,745,600-20,338,360)x1	3.5	Smith-Magenis syndrome	P	TOP
110	35	VSD,AMA	arr[hg19] 17p11.2(16,763,698-20,457,631)x1	3.69	Smith-Magenis syndrome	P	TOP
111	39	AMA	arr[hg19] 17p12(14,087,918-15,473,312)x3	1.38	Charcot-Marie-Tooth disease type 1A	P	TOP
112	27	High risk maternal serum screening	arr[hg19] 17p12(14,087,919-15,491,109)x1	1.4	Hereditary neuropathy with liability to pressure palsies	P	live born
113	30	Requirements for pregnant women	arr[hg19] 17p12(14,060,336-15,484,335)x1	1.42	Hereditary neuropathy with liability to pressure palsies	P	live born
114	33	High risk maternal serum screening	arr[hg19] 17p12(14,073,536-15,503,234)x1	1.4	Hereditary neuropathy with liability to pressure palsies	P	TOP
115	26	NIPT(+)	arr[hg19] 18q22.1q23(64,084,774-78,013,728)x3 arr[hg19] 12p13.33(173,786-1,416,241)x1	13.9/1.2	8q22.1q23 duplication/12p13.33 deletion	LP	TOP

116	40	NIPT(+),AMA	arr[hg19] 18p11.32p11.21(136,227-15,099,116)x1	14.9	Holoprosencephaly 4	P	TOP
117	32	NT(+)	arr[hg19] 18p11.32p11.21(136,228-15,099,116)x1<br>arr[hg19] 5p15.33p14.1(113,577-26,482,324)x3	15.0/26.4	Holoprosencephaly 4/5p15.33p14.1duplication	P	TOP
118	38	NIPT(+)， AMA	arr[hg19] 18p11.32p11.21(136,227-15,181,208)x3	15.04	18p11.32p11.21 duplication	P	TOP
119	34	Pericardial effusion，aberrant right subclavian artery	arr[hg19] 18q21.2q23(50,313,881-78,013,728)x1	27.7	18q terminal deletions	P	TOP
120	36	NIPT(+),AMA	arr[hg19] 18q22.1q23(63,734,464-78,013,728)x1	14.2	18q terminal deletions	P	TOP
121	28	NIPT(+)	arr[hg19] 18q22.3q23(70,367,252-78,013,728)x1	7.6	18q terminal deletions	P	TOP
122	35	NIPT(+),AMA	arr[hg19] 18q22.2q23(67,504,998-78,013,728)x1	10.51	18q terminal deletions	P	TOP
123	18	NIPT(+)	arr[hg19] 18q21.32q23(58,703,880-78,013,728)x1	19.3	18q terminal deletions	P	TOP
124	32	NIPT(+)	arr[hg19] 18q22.1q23(62,751,865-78,013,728)x1<br>arr[hg19] 12p13.33p13.2(173,786-11,663,677)x3	15.2/11.5	18q terminal deletions/Pallister-Killian syndrome	P	TOP

125	28	High risk maternal serum screening	arr[hg19] 21q21.1q22.3(18,052,901-48,093,361)x3 arr[hg19] Xp22.33p11.4(168,551-41,819,195)x1	30/41	Down syndrome	P	TOP
126	30	NIPT(+)	arr[hg19] 22q11.21(18,919,477-21,800,471)x3	2.88	22q11.2 duplication syndrome	P	TOP
127	27	NIPT(+)	arr[hg19] 22q11.21(18,648,855-21,461,017)x3	2.8	22q11.2 duplication syndrome	P	TOP
128	26	NIPT(+)	arr[hg19] 22q11.21(18,648,855-21,459,713)x3	2.8	22q11.2 duplication syndrome	P	TOP
129	24	VSD	arr[hg19] 22q11.21(18,919,477-21,459,713)x3	2.54	22q11.2 duplication syndrome	P	TOP
130	35	NIPT(+),AMA	arr[hg19] 22q11.21(18,648,856-21,800,471)x3	3.15	22q11.2 duplication syndrome	P	TOP
131	41	AMA	arr[hg19] 22q11.21(18,648,856-21,800,471)x3	3.15	22q11.2 duplication syndrome	P	TOP
132	28	NIPT(+)	arr[hg19] 22q11.1q11.21(16,888,899-21,800,471)x3	4.91	22q11.2 duplication syndrome	P	TOP
133	38	NIPT(+),AMA	arr[hg19] 22q11.21(18,648,855-21,800,471)x3	3.15	22q11.2 duplication syndrome	P	TOP

134	27	NIPT(+)	arr[hg19] 22q11.21(18,648,855-21,800,471)x3	3.15	22q11.2 duplication syndrome	P	live born
135	25	NIPT(+)	arr[hg19] 22q11.21(18,636,749-21,800,471)x1	3.2	DiGeorge /Velocardiofacial syndrome	P	TOP
136	30	Foot varus，Left ventricular punctate strong echo	arr[hg19] 22q11.21(18,648,855-21,800,471)x1	3.15	DiGeorge /Velocardiofacial syndrome	P	TOP
137	20	Right aortic arch	arr[hg19] 22q11.21(18,916,843-21,800,471)x1	2.88	DiGeorge /Velocardiofacial syndrome	P	TOP
138	26	Adverse pregnancy	arr[hg19] 22q11.21(18,919,478-20,312,661)x1	1.39	DiGeorge /Velocardiofacial syndrome	P	TOP
139	24	NIPT(+)	arr[hg19] 22q11.21(18,648,856-21,800,471)x1	3.15	DiGeorge /Velocardiofacial syndrome	P	TOP
140	30	NT(+)	arr[hg19] 22q11.1q11.21(16,888,899-18,649,190)x4	1.76	Cat-Eye-Syndrome	P	TOP
141	31	NT(+)	arr[hg19] 22q13.31q13.33(46,929,980-51,197,766)x1 arr[hg19] 12q24.31q24.33(123,650,000-133,777,562)x3	4.2/10.1	Phelan-McDermid Syndrome	P	TOP
142	25	NIPT(+)	arr[hg19] Xp11.21q28(56,754,945-155,233,098)x3 arr[hg19] Xp22.33p11.21(168,551-56,754,782)x1	98/56	Isodicentric X chromosome	P	TOP

143	24	Requirements for pregnant women	arr[hg19] Xp22.31(6,455,151-8,135,568)x0	1.7	X-linked Ichthyosis	P	live born
144	30	High risk maternal serum screening	arr[hg19] Xp22.31(6,455,151-8,135,568)x0	1.7	X-linked Ichthyosis	P	live born
145	34	NIPT(+)	arr[hg19] Xp22.31(6,455,152-8,135,568)x0	1.7	X-linked Ichthyosis	P	live born
146	30	NIPT(+)	arr[hg19] Xp22.33p22.2(168,551-15,116,848)x1 arr[hg19]10q25.1q26.3(111,312,762-135,426,386)x3	14.9/24.1	Xp22.33p22.2 deletion/10q25.1q26.3 duplication	P	TOP
147	32	NIPT(+)	arr[hg19] Xp22.33p21.1(168,551-33,349,792)x1	33.18	Xp22.33p21.1 deletion	P	TOP
148	34	NIPT(+)	arr[hg19] Xp22.33p11.3(168,552-46,035,191)x1	45.86	Xp22.33p11.3 deletion	P	TOP
149	32	High risk maternal serum screening	arr[hg19] Xp22.33p21.2(168,552-30,298,754)x1	30.1	Xp22.33p21.2 deletion	P	TOP
150	30	VSD	arr[hg19] Xp22.33 (168,552-1,734,398)x1	1.56	Leri-Weill dyschondrosteosis	P	TOP
151	34	NIPT(+)	arr[hg19] Xp22.33p22.31(168,552-7,535,782)x1 arr[hg19]Yq11.21q11.23(14,994,328-28,799,654)x1	7.36/13.8	Xp22.33p22.31 deletion	P	live born

152	34	NIPT(+)	arr[hg19] Xp22.33p11.3(168,552-44,075,667)x1 arr[hg19]17q23.2q25.3(58,507,547-81,041,823)x3	43.9/23.5	Xp22.33p11.3 deletion /17q23.2q25.3 duplication	P	TOP
153	35	NIPT(+),AMA	arr[hg19] Xp11.23p11.22(48,169,055-52,685,151)x1	4.51	Xp11.23p11.22 deletion	P	TOP
154	30	Adverse pregnancy	arr[hg19] Xp21.1(31,795,775-31,962,949)x0	0.167	Duchenne muscular dystrophy/Becker muscular dystrophy	P	live born
155	36	NIPT(+),AMA	arr[hg19] Xp22.33p21.2(415,997-29,929,100)x2	29.5	Xp22.33p21.2 duplication	P	TOP
156	26	NIPT(+)	arr[hg19] Xq21.31q28(88,050,067-155,233,098)x1	67	Xq terminal deletion	P	TOP
157	27	NIPT(+)	arr[hg19] Xq22.1q28(102,451,933-155,233,098)x1	52.7	Xq terminal deletion	P	TOP
158	27	IUGR	arr[hg19] 5q23.2q35.3(121,401,139-180,715,096)x3 arr[hg19] Xq23q28(115,956,088-155,233,098)x1	59/39	Xq terminal deletion/5q23.2q35.3duplication	P	TOP
159	28	Bipedal hexadactylia	arr[hg19] Xq21.1q21.33(79,432,014-95,863,272)x1	16.4	Xq21.1q21.33 deletion	P	TOP

Schedule 2: Uncertain significance copy number variations found in fetuses with indication for genetic study by CMA

Case	Age	Clinical indications	CMA Results [hg19]	Size (Mb)	Interpretation	Outcome
1	33	Requirements for pregnant women	arr[hg19] 13q13.1q13.2(32,636,393-35,307,066)x1	2.67	13q13.1q13.2 deletion	live born
2	24	High risk maternal serum screening	arr[hg19] Xp22.33q28(169,921-155,233,846)x2 hmz	155	ROH	live born
3	22	NIPT(+)	arr[hg19] 8q24.12q24.13(119,362,072-124,561,355)x3	5.2	8q24.12q24.13duplication	live born
4	30	CPC	arr[hg19] 18q22.1q22.2(66,328,070-67,710,537)x3	1.38	18q22.1q22.2duplication	live born
5	21	IUGR	arr[hg19] 2p23.1(30,677,219-31,949,181)x1	1.27	2p23.1deletion	TOP
6	34	Ectopic right kidney	arr[hg19] 22q11.23(23,652,517-25,002,659)x3	1.35	22q11.23 duplication	TOP
7	31	NT(+)	arr[hg19] 2p11.2(86,601,136-88,134,752)x1	1.53	2p11.2 deletion	Refuse
8	22	Adverse pregnancy	arr[hg19] 22q11.21q11.22(21,460,640-23,221,864)x3	1.76	22q11.21q11.22duplication	live born

9	29	Adverse pregnancy	arr[hg19] 1p13.2p12(115,765,487-119,134,297)x1	3.36	1p13.2p12 deletion	TOP
10	29	High risk maternal serum screening	arr[hg19] Xp22.33p22.2(482,483-16,768,717)x2<br>arr[hg19] Yp11.31q11.223(2,650,424-22,243,236)x2	16.28/19.59	Xp22.33p22.2 duplication /Yp11.31q11.223duplication	TOP
11	38	AMA	arr[hg19] 4p16.3q35.2(75,173-190,921,709)x2 hmz	190.8	ROH	TOP
12	29	NT(+)	arr[hg19]10q11.22q11.23(46,252,072-51,903,756) x 3	5.65	10q11.22q11.23 duplication	live born
13	21	Requirements for pregnant women	arr[hg19]10p12.31p12.2(21,836,123-23,345,632)x1	1.51	10p12.31p12.2 deletion	live born
14	30	Requirements for pregnant women	arr[hg19]10q11.22q11.23(46,981,524-52,550,240)x3	5.56	10q11.22q11.23duplication	live born
15	27	NIPT(+)	arr[hg19] 15q26.3(100,074,926-102,429,040)x1	2.35	15q26.3duplication	TOP
16	39	AMA	arr[hg19]15q23q26.1(68,640,383-93,807,986) hmz	25.16	ROH	TOP
17	42	AMA	arr[hg19] 11q13.3(68,458,754-69,550,591)x3	1.09	11q13.3duplication	TOP

18	30	NIPT(+)	arr[hg19] 7q36.3(158,393,513-159,119,707)x1	0.73	7q36.3 deletion	live born
19	28	IUGR	arr[hg19] 3q26.32(177,717,453-178,906,429)x3	1.18	3q26.32 duplication	live born
20	24	NIPT(+)	arr[hg19] 7q31.1(110,409,106-110,742,088)x1	0.33	7q31.1 deletion	TOP
21	30	Venous catheter α Wave reversal	arr[hg19] 22q11.21(18,648,855-19,267,194)x1	0.62	22q11.21 deletion	TOP
22	30	High risk maternal serum screening	arr[hg19] 5q33.2(152,967,794-155,406,427)x1	2.43	5q33.2 deletion	TOP
23	26	Venous catheter α Wave reversal	arr[hg19]2q12.2q12.3(106,873,992-108,527,327)x1	1.65	2q12.2q12.3 deletion	live born
24	25	Adverse pregnancy	arr[hg19]2q14.2q14.3(121,957,242-126,703,771)x1	4.7	2q14.2q14.3deletion	live born
25	33	NIPT(+)	arr[hg19]16p13.3p12.3(94,807-19,331,243)x2hmz	19.24	ROH	live born
26	26	NT(+)	arr[hg19] 22q11.23(23,653,979-25,041,592)x3	1.38	22q11.2 duplication	live born

27	24	NIPT(+)	arr[hg19]13q31.1q31.3(81,828,141-92,829,200)x3	11	13q31.1q31.3duplication	live born
28	27	NIPT(+)	arr[hg19]22q11.22q11.23(22,997,928-25,0002659)x3	2.2	22q11.2 duplication	live born
29	16	NIPT(+)	arr[hg19] 6p12.1q13(55,523,793-73,419,735)x3<br>arr[hg19]6q16.1q16.3(96,588,337-103,062,754)x3	17.89/6.47	6p12.1q13duplication<br>/6q16.1q16.3 duplication	TOP
30	28	EB(+)	arr[hg19] 22q11.21(21,058,887-21,800,471)x1	0.74	22q11.21deletion	TOP
31	29	Adverse pregnancy	arr[hg19]21q22.12q22.13(36,489,368-37,849,706)x3	1.36	21q22.12q22.13 duplication	live born
32	34	Requirements for pregnant women	arr[hg19] 16p11.2(28,786,703-29,032,280)x3	0.25	16p11.2 duplication	live born
33	40	NIPT(+)	arr[hg19] 8p23.3p23.2(158048-4,289,942)x1	4.12	8p23.3p23.2 deletion	TOP
34	40	AMA	arr[hg19] 11p15.2(14,487,525-15,584,061)x3<br>arr[hg19] 11q25(133,609,389-134,336,032)x1	1.09/0.72	11p15.2 duplication<br>/11q25deletion	TOP
35	45	NIPT(+)	arr[hg19] 15q25.1q26.3(80,932,216-102,397,317) hmz	21.45	ROH	TOP

36	22	NIPT(+)	arr[hg19] 2p25.3(271,525-4,093,110)x3	3.82	2p25.3 duplication	TOP
37	30	High risk maternal serum screening	arr[hg19] 2q13(110,498,141-110,980,295)x1	0.48	2q13 deletion	live born
38	29	Adverse pregnancy	arr[hg19] 19q13.2(40,933,980-42,057,333)x3	1.12	19q13.2 duplication	TOP
39	34	Duplicated kidney	arr[hg19]15q25.2q25.3(84,915,505-85,723,137)x3	0.81	15q25.2q25.3duplication	live born
40	17	High risk maternal serum screening	arr[hg19] 21q22.3(47,692,008-48,093,361)x1	0.4	21q22.3 deletion	live born
41	22	Aberrant right subclavian artery	arr[hg19] 1p31.1(73,814,103-74,844,603)x1	1.03	1p31.1 deletion	TOP
42	22	aberrant right subclavian artery	arr[hg19] 16q22.1(67,055,940-69,223,727)x3	2.16	16q22.1 duplication	live born
43	39	AMA	arr[hg19] 22q11.23(23,653,980-25,041,592)x3	1.38	22q11.23 duplication	live born
44	33	High risk maternal serum screening	arr[hg19]Yq11.221q11.223(19,571,467-25,863,576)x0	6.29	Yq11.221q11.223 deletion	live born

45	42	AMA	arr[hg19] 20p12.3p12.2(8,590,883-9,474,261)x1	0.88	20p12.3p12.2 deletion	live born
46	22	NIPT(+)	arr[hg19] 2p25.3p23.2(12,771-29,922,364)x3[0.15]arr[hg19]6q22.31q27(123,468,206-170,914,297)x3[0.13]	29.9/47.7	2p25.3p23.2 mosaic duplication /6q22.31q27 mosaic duplication	live born
47	17	VSD	arr[hg19]8q13.3q22.3(70,625,942-103,705,765)x2 hmz arr[hg19]8q24.12q24.3(121,072,939-146,292,734)x2 hmz	33.08/25.22	ROH	live born
48	21	NT(+)	arr[hg19] 15q13.3q14(32,444,261-34,671,601)x3	2.22	15q13.3q14 duplication	TOP
49	31	Complete obstruction of the upper end of the jejunum	arr[hg19]2q22.2q32.1(143,194,361-186,185,653)x2 hmz arr[hg19] 18q12.2q22.3(35,056,608-69,357,603)x2 hmz arr[hg19] 13q32.3q34(101,185,001-115,095,705)x2 hmz	42.9/ 32.3/13.9	ROH	TOP
50	24	Right foot varus	arr[hg19] 6p21.2(38,794,107-39,919,544)x3	1.12	6p21.2 duplication	live born
51	38	AMA	arr[hg19] 4q35.2(189,399,585-190,957,460)x1	1.55	4q35.2 deletion	TOP
52	29	NIPT(+)	arr[hg19]14q21.1q21.2(38,016,666-46,778,047)x1	8.76	14q21.1q21.2 deletion	TOP
53	33	NIPT(+)	arr[hg19] 5p15.33p14.1(113,577-27,967,386)x2 hmz	27.85	ROH	live born

54	31	NIPT(+)	arr[hg19]8p23.1p11.1(8,117,565-43,776,564)x2 hmz	35.65	ROH	live born
55	39	AMA	arr[hg19] 7p22.1(4,620,799-5,828,233)x3	1.2	7p22.1 duplication	live born
56	32	High risk maternal serum screening	arr[hg19] 4q35.2(187234366_190742076)x1	3.5	4q35.2 deletion	live born
57	35	High risk maternal serum screening	arr[hg19] 18q22.1(62,175,841-65,692,392)x1	3.51	18q22.1 deletion	live born
58	32	NIPT(+)	arr[hg19] 2p24.3p24.2(13,619,816-18,973,924)x3	5.35	2p24.3p24.2 duplication	live born
59	21	VM(+)	arr[hg19]9q34.11q34.13(132,977,865-135,767,483)x1	2.79	9q34.11q34.13duplication	live born
60	35	NIPT(+)	arr[hg19]4q35.1q35.2(186,846,098-190,806,055)x1	3.96	4q35.1q35.2 deletion	TOP
61	35	NIPT(+)	arr[hg19] Xp22.33(1,906,014-2,992,586)x3	1.08	Xp22.33 duplication	TOP
62	35	NIPT(+)	arr[hg19] 5p15.33(113,577-4,012,797)x1	3.9	5p15.33 deletion	TOP

63	34	High risk maternal serum screening	arr[hg19] 1q21.1(144,567,360-145,775,966)x1	1.2	1q21.1deletion	TOP
64	31	NIPT(+)	arr[hg19] Xp22.33 (168,552-995,018)x1	0.82	Xp22.33 deletion	live born
65	35	NIPT(+)	arr[hg19]21q21.1q21.2(19,270,208-25,089,781)x3	5.82	21q21.1q21.2 duplication	live born
66	32	High risk maternal serum screening	arr[hg19] 10q26.3(133,926,647-135,426,386)x3	1.5	10q26.3 duplication	TOP
67	29	Aberrant right subclavian artery	arr[hg19] 17q25.1(73,319,633-74,385,752)x1	1.06	17q25.1 deletion	live born
68	34	High risk maternal serum screening	arr[hg19]12q11q14.3(38,190,103-66,156,098)x2 hmz arr[hg19] 8p23.1p12(8,117,565-29,922,009)x2 hmz	27.9/21.8	ROH	live born(Six fingers of the right hand)
69	36	NIPT(+)	arr[hg19]4q28.1q28.3(127,947,800-138,415,471)x3	10.46	4q28.1q28.3 duplication	live born
70	26	High risk maternal serum screening	arr[hg19]4q34.3q35.1(181,956,790-183,645,544)x1	1.68	4q34.3q35.1 deletion	live born
71	34	NIPT(+)	arr[hg19]10q26.13q26.3(123,468,677-135,426,384)x2 hmz	11.95	ROH	live born

72	37	AMA	arr[hg19] Xq26.2(130,695,165-131,646,711)x2	0.95	Xq26.2 duplication	TOP
73	35	Adverse pregnancy	arr[hg19] 15q21.2(51,225,021-51,910,044)x4	0.68	15q21.2 duplication	TOP
74	47	AMA	arr[hg19] 21q22.3(47,150,415-48,093,361)x1	0.94	21q22.3 deletion	live born
75	29	High risk maternal serum screening	arr[hg19]10q26.13q26.2(127,445,240-129,359,361)x3	1.91	10q26.13q26.2 duplication	live born
76	35	AMA	arr[hg19] 17q11.2(29,004,605-30,341,286)x3	1.33	17q11.2 duplication	live born

Appendix :

CMA: Chromosomal microarray analysis

CNVs: Copy Number Variants

NIPT: Non-invasive Prenatal Testing

AMA: Advanced Maternal Age

MSS: Maternal Serum Screening

ROH: Regions of Homozygosity

NT: Nuchal Thickness

VSD: Ventricular Septal Defect

IUGR: Intrauterine Growth Retardation

VM: Ventriculomegaly

PLSVC: Persistent Left Superior Vena Cava

SUA: Single Umbilical Artery;

EB: Echogenic bowel

TOP: Terminations of Pregnancy
